# Effects of tag type and surgery on migration of Atlantic salmon (*Salmo salar*) smolts

**DOI:** 10.1111/jfb.15116

**Published:** 2022-06-27

**Authors:** Robert J. Lennox, Elisabeth Stöger, Lotte S. Dahlmo, Turid Helle, Tore Wiers, Erlend M. Hanssen, Knut Wiik Vollset

**Affiliations:** ^1^ NORCE Norwegian Research Centre Laboratory for Freshwater Ecology and Inland Fisheries Bergen Norway

## Abstract

Tagging salmon smolts to provide information about the timing of outmigration has been a common approach to monitor phenology and model the risk of encountering stressors. However, the validity of tagging has come under scrutiny because of the sensitivity of this parameter in various management systems. We studied the probability of migration, timing of migration and growth during migration for Atlantic salmon smolts tagged with three different tags in the River Dale, western Norway. Two groups were tagged with passive integrated transponder (PIT) tags *via* a small ventral nonsurgical incision, either a 12 mm or a new 16 mm PIT tag. Two groups were subjected to surgical implantation of either a dummy acoustic transmitter or a 12 mm PIT tag (a sham surgery). Overall, 71% of the tagged smolts were recaptured at the downstream Wolf trap. Smolts from the sham tagged group were recaptured most frequently (78%) compared to dummy acoustic transmitters and 16 mm PIT tags (both 68%), but the differences were not significant. Results agree with prior assessments that longer smolts migrated earlier, with about half a day earlier migration for each millimetre total length of the smolt, but did not suggest any difference in time of migration among the tag types. Growth in length was evident from release to recapture, with smaller smolts exhibiting greater growth and no effect of tagging treatment. Our findings suggest that inferences about the timing of outmigration for salmon smolts based on acoustic tagging should be made cautiously because of the relationship among tag size, suitable fish size and the timing of a tagged individual's migration.

## 1 INTRODUCTION

Electronic tags are relied on for gathering data on the life history and behaviour of animals in the context of environmental monitoring (Hussey *et al*., 2015). Various tags and tagging methods are available, which must be scrutinized to select the best option for a study or monitoring programme. For anadromous species, passive integrated transponder (PIT) tags and acoustic transmitters are frequently used to tag and track the fate of fish as they move up‐ or downriver. PIT tags are marketed in various sizes but are generally small because they do not carry a battery and instead communicate their unique identification number across a short distance when charged by an electronic reader (Prentice, 1990). Acoustic transmitters are battery powered and are larger than PIT tags, but have a limited battery life (Voegeli *et al*., 1998). Nevertheless, acoustic transmitters are preferred for certain applications that require more detailed observation of the individual animals. The larger acoustic tags also require a surgical implantation *via* insertion through a ventral incision and closure of the wound site under anaesthesia (Jepsen *et al*., 2002), whereas PIT tags can be inserted more rapidly through a small puncture in the ventral cavity, typically also under anaesthesia.

Electronic tagging of salmonid smolts has formed the basis of global monitoring programmes informing hydropower (Havn *et al*., 2018; Renardy *et al*., 2020), impacts of pathogens (Lennox *et al*., 2020), aquaculture environmental interactions (Rechinsky *et al*., 2021; Vollset *et al*., 2017) and more. Using tagging studies to provide reliable data on the timing of outmigration of salmon smolts has come under particular scrutiny in recent years (Hulbak *et al*., 2021). The timing of outmigration has been important for management in various contexts, for example to assess how to best regulate operations of hydropower (Alfredson *et al*., 2012), but has been made even more relevant as studies suggest that modelling the impact of salmon lice on the survival of outmigrating Atlantic salmon (*Salmo salar* Linnaeus 1758) is very sensitive to correctly describing the timing of outmigration of salmon smolt (Torrissen *et al*., 2013). A clear interest in correctly describing this sensitive parameter has spurred a series of studies using various methods, including telemetry tags (Bass *et al*., 2020; Newton *et al*., 2016; Vollset *et al*., 2020; Welch *et al*. 2007). A concrete criticism against using tagging rather than noninvasive monitoring methods is that (1) the handling, tag burden and tagging may impact the outmigration time and survival of the tagged fish, and (2) that there may be selection when capturing and selecting fish to tag that may bias the results. Even so, in most systems, noninvasive methods such as cameras are not feasible and require tagging methods to study the outmigration of salmon. When data are needed about salmon smolt migrations to monitor potential exposure to stressors, tagging is often the best method available to generate accurate results, but the relevant limitations must be well established and understood for informed decision‐making based on the results.

In this study, we investigated how different tagging procedures and tag types affected the migration and growth of wild smolts in a regulated river. We sorted Atlantic salmon smolts into four tagging groups to investigate the effects of different tagging options for tracking their riverine migration. Following capture and anaesthesia, fish were tagged with small or medium‐length PIT tags or underwent surgery. In one surgery group, fish were implanted with a dummy acoustic tag encapsulating a PIT tag and in the second group only a small 12 mm PIT tag was inserted as a sham surgery. Recapture of the tagged fish and individual identification from the PIT ID permitted an assessment of the migration, timing and growth of the smolts from the four groups.

## 2 MATERIALS AND METHODS

### 2.1 Study site

The study was conducted in the Dale River located in Vaksdal municipality, a 4.7 km river in western Norway that drains into the Osterfjord. This is a regulated river, impacted by hydropower in several steps since 1927. A Wolf trap installed in the river is used to enumerate smolts migrating out from the area upstream of the hydropower outlet annually and allows an opportunity to recapture smolts on their seaward migration (Hulbak *et al*., 2021). The Wolf trap covers the entire length of the river and is assumed to capture all downstream migrating fish, although mark‐recapture estimates from previous years suggest that some evade capture, potentially during high flow events (Hulbak *et al*., 2021).

### 2.2 Fish capture

Atlantic salmon smolts were captured by backpack electrofishing in the Dale River above the Wolf trap in spring 2021. Three hundred and forty‐one smolts were tagged; there were 50 dummy tagged smolts, 51 sham tagged smolts, and 120 tagged with 12 mm and 120 with 16 mm PIT transmitters implanted by a small nonsurgical incision. Tagged Atlantic salmon smolts migrating in the Dale River were recaptured in the Wolf trap, about 200–300 m downstream of the tagging site. Previous studies have confirmed that smolts moving from this area to the trap are migrating based on osmoregulatory Adenosine triphosphate‐ase activity (Hulbak *et al*., 2021). The Wolf trap was operational in the springtime during the smolt run from March 21 to June 23, 2021. The trap was attended daily to scan and release captured fish.

### 2.3 Instrumentation

Dummy transmitters were manufactured by Thelma Biotel (Trondheim, Norway) to emulate the size and weight of the standard 6 mm acoustic transmitter (14.5 × 6.3 mm, 1.2 g in air) with a 12 mm PIT tag (12.50 × 2.12 mm, 0.1 g in air; Biomark, Boise, USA) encapsulated inside the tag so that it could be detected if recaptured. A true control and true sham were not possible in the field setting where we needed to redetect the fish, so we conducted a sham acoustic surgery with a small (12 mm) PIT tag inserted into the body cavity through the surgical incision. All fish were anaesthetized in tricaine methanesulfonate (0.1 g l^−1^) buffered with sodium biocarbonate (0.1 g l^−1^). Surgeries for treatment and sham tagged fish involved transfer of the fish from anaesthetics to a tagging cradle, where the fish was placed supine with half‐dose anaesthetic water piped into the mouth and over the gills. The tag was implanted through an incision made with a surgical scalpel and closed with two interrupted sutures (4/0 Ethilon Vicryl suture). The surgery groups were compared to two PIT tag groups for which either the 12 mm or 16 mm (16 × 3 mm, 0.25 g in air; RFID Solutions, Stavanger, Norway) PIT tag was inserted using standard implantation methods, through a small nonsurgical incision made with a surgical scalpel in <10 s. Minimum fish lengths for tagging were 129 mm for the dummy tag, 124 mm for the sham procedure, 103 mm for the 12 mm PIT tag and 110 mm for the 16 mm PIT tag.

### 2.4 Data analysis

#### 2.4.1 Probability of migration to the wolf trap

To test if the treatment had an effect on the probability of migration, the smolts were categorized as migratory or nonmigratory based on whether or not they were redetected in the Wolf trap. Migration was then analysed using a generalized linear model with the *glm* function in R. Considering we had a limited sample size, we conducted a simulation to estimate statistical power. The power analysis assumed 50 fish tagged per treatment of equal length and used the *rbinom* function to draw 1s or 0s from a binomial distribution. The probability of drawing a 1 was set to be 80% for 12 mm PIT, 16 mm PIT and sham treated fish, and 70% for dummy tagged fish to assess the probability of identifying a significant effect of dummy tagging given a true 10% difference in migration to the Wolf trap between dummy tags and counterparts. The percentage of times out of 10,000 simulations the dummy treatment was significant in a logistic regression relative to the baseline (12 mm PIT) was assumed to be the test's power to detect a true 10% difference (see Appendix S1).

A logistic regression was used to test for an effect of length and treatment on migration probability. To make multiple comparisons among the four tagging groups, a Tukey test was implemented using the *glht* function in the R package multcomp.

#### 2.4.2 Effects of tagging on of time of migration

The number of days to outmigration was calculated from the difference between capture and recapture date, and analysed with a linear regression model using the *lm* function. Multiple comparisons were performed with the Tukey test.

#### 2.4.3 Growth during migration

Individual growth was calculated based on the initial fish length (total length) and the length upon recapture in the Wolf trap. A simple linear model was fit to explain the change in length of individuals as a function of treatment group and time interval between capture and recapture. Initial length was also included in an attempt to control for potential differences associated with fish size at the outset of the study.

## 3 RESULTS

Three hundred and forty‐one Atlantic salmon smolts were tagged in the Dale River: 50 dummy acoustic tags (139 ± 6 s.d. mm total length), 51 sham surgeries (138 ± 7 s.d. mm total length) and 120 each of 12 mm (124 ± 10 s.d. mm total length) and 16 mm PIT tags (127 ± 8 s.d. mm total length). Average tag burden for dummy tagged fish was 5.8% of body weight, 0.14% for 16 mm PIT, 0.06% for 12 mm PIT and 0.05% for the sham. Among the tagged smolts, 70% (*N* = 240) were recaptured in the Wolf trap during a 43 day period. In total, 5965 untagged salmon were caught during the same period (147 ± 43 s.d. mm total length).

### 3.1 Probability of migration to the Wolf trap

**FIGURE 1:**
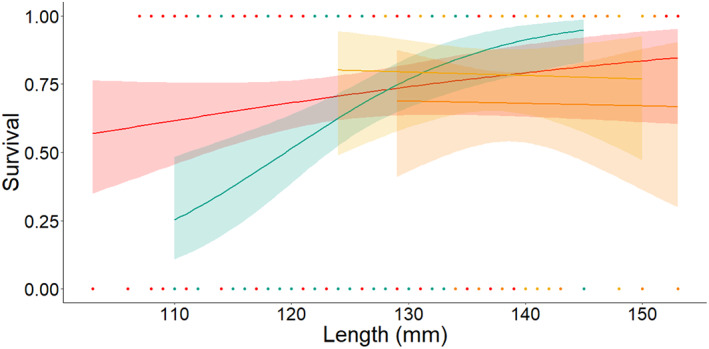
Simple logistic curves explaining the proportion of Atlantic salmon (*Salmo salar*) smolts recaptured in the Dale River Wolf trap. Smolts tagged with 16 mm PIT and dummy acoustic transmitters had the lowest overall rates of detection (both 68%). There was a significant size effect on detection but no differences among treatments. 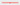 HDX‐12 mm; 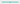 HDX‐16 mm; 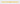 sham; 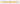 dummy

Smolts in the sham group were detected most frequently (78%), followed by 12 mm PIT tag (71%), dummy acoustic transmitter (68%) and 16 mm PIT tag (68%). The power analysis suggested that the logistic regression would detect a 10% true difference in smolt migration between the dummy tag and the 12 mm PIT tag 20% of the time, and a 20% difference 58% of the time. Length had a significant effect on smolt migration (*z* = 3.01, *P* < 0.01). Odds of migration increased by 1.04 per millimetre of total length. Tag type did not have a significant effect on the migration (all Tukey HSD *z* values were less than 1.85 and all *P* values were greater than 0.24). Length did not have a significant effect on migration when the length minimum was set to 125 mm (*z* = 0.78, *P* = 0.44), and nor did tag type (all Tukey HSD *z* values were less than 1.63 and all *P* values were greater than 0.36; Figure 1).

### 3.2 Effects of tagging on time of migration

**FIGURE 2:**
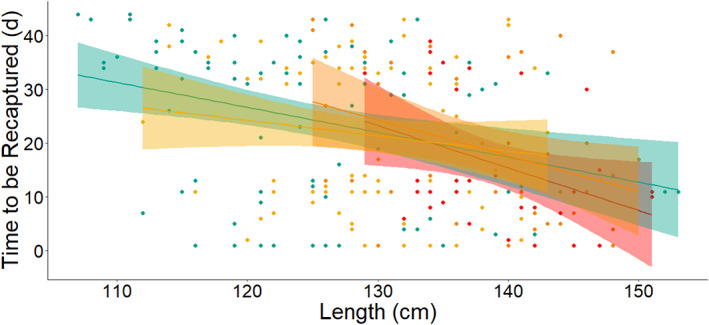
Days to be recaptured for the four treatment groups as a function of length at tagging. 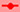 dummy; 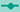 HDX‐12 mm; 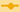 HDX‐16 mm; 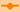 sham

The linear regression showed there was a significant effect of length on migration time interval across all tag groups (Figure 2). There was a negative relationship between migration interval and length, with longer smolts migrating earlier than shorter smolts. Each millimetre of total length reduced the days to migrate by 0.47 days. There were no significant differences among the tag types (all Tukey HSD *t* values were less than 0.77 and all *P* values were greater than 0.86).

### 3.3 Growth during migration

**FIGURE 3:**
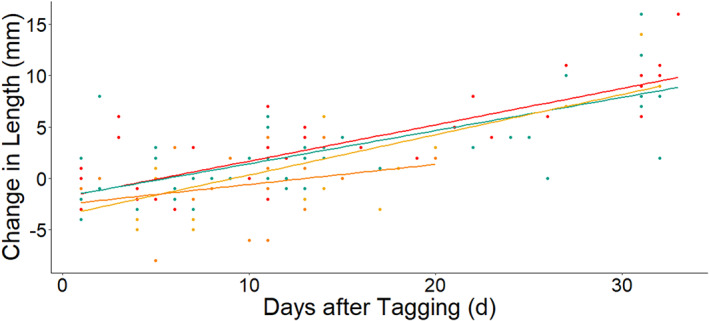
Growth intervals of Atlantic salmon smolts captured in the Wolf trap in the Dale River. 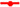 HDX‐12 mm; 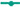 HDX‐16 mm; 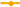 sham; 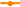 dummy

The dummy tagged smolt growth (−0.75 ± 3.10 s.d. mm) was less than, but not significantly different from, growth of the 12 mm PIT tag group (12 mm: 2.91 ± 4.68 s.d. mm, *t* = 1.97, *P* = 0.07) nor the 16 mm PIT tag group (2.56 ± 4.47 s.d. mm, *t* = 1.55, *P* = 0.12). Growth was also not different between the dummy tag and sham (1.10 ± 4.33 s.d. mm) treatment (*t* = 1.03, *P* = 0.30). The effect of initial length was nearly significant (*t* = −1.97, *P* = 0.05), with a negative slope suggesting slower growth for larger smolts. Negative average growth by the dummy tagged group suggests some measurement error and not actually shrinking of fish. The time interval was also significant (*t* = 12.96, *P* < 0.01) such that smolts were expected to grow about 0.34 mm per day after tagging (slope; Figure 3).

## 4 DISCUSSION

We found no significant effects of tag types on the probability of migration or migration timing of Atlantic salmon smolts in the Dale River. Surgical procedures tend to be viewed as more invasive than a small incision to insert a miniature PIT tag, which is much smaller than an acoustic tag because it does not carry a battery on board and is instead charged by an external power source to transmit the tag‘s identification code. However, we found that Atlantic salmon smolts migrated at similar rates regardless of the tag treatment and that instead length was a strong indicator of the timing of migration, aligning with Hulbak *et al*. (2021) conducted in the same system. The results suggest that investigators can use electronic tags suitable for the size classes of their target and smolts up to ~5% of body weight without significant effects on the migration compared to less invasive methods.

Most (70%) of the smolts tagged in this study migrated prior to the removal of the Wolf trap on June 23, a period of 54 days. Newton *et al*. (2016) recorded 60% of large smolts reaching the sea in their study, although the migration distance was several orders of magnitude longer. Smolts that were not detected may have been eaten by local crows (*Corvus cornix*) that were observed catching smolts in the Dale River or by other predators. Smolts may also have died as a consequence of capture/handling/tagging, may have postponed migration for a future season or year, or may have passed the Wolf trap undetected. According to Birnie‐Gauvin *et al*. (2019), autumn migration of smolts can be an important subset of the overall migration, but is rarely well documented; the Wolf trap is absent in the autumn so we had no ability to detect this. Hulbak *et al*. (2021) estimated that 29% of undetected fish passed the same Wolf trap in 2019, suggesting that 30 of the 102 (29%) undetected smolts from our study might have passed the Wolf trap undetected this year as well. It is unlikely that many of the smolts died from capture/handling/tagging; laboratory results of tagging have shown extremely high rates of post‐tagging survival. If some fish did die, it seems that there were equal numbers of mortalities among groups given only weak evidence that the dummy tagged acoustic fish had lower detection probability than the 12 mm PIT tagged fish. The significant effect of fish length on migration suggests that smaller fish were less likely to be detected and were delaying migration for a future year due to their small size. If there was size‐dependent predation such that small fish were more likely to be taken by predators, our ability to resolve size effects in the migration data may be biased. However, Hostetter *et al*. (2012) showed that steelhead (*Oncorhynchus mykiss*) smolt vulnerability to predation increased with body length up to 202 mm; if this were the case, we would expect an even more extreme effect of small size on migration of these salmon smolts.

Migration timing was related to length of the smolts at tagging with about half a day of delay for each millimetre of total length, suggesting longer fish migrated early. Migration timing is size‐dependent in Atlantic salmon smolts and Hulbak *et al*. (2021) found that smaller smolts grew during the season and migrated later than larger counterparts. We found nearly identical results such that size at tagging was a significant predictor of migration delay. This has implications for tagging studies because of size limitations for acoustic transmitters such that the smallest individuals in a cohort, which will migrate later based on our results, will not be tagged with acoustic transmitters. The consequence of this is that the smolt migration window will appear to be shorter and earlier when acoustic tagged fish are used to model the outmigration timing. Tagging smaller fish that migrate later is crucial for studies to effectively capture the full extent of the smolt migration window. The modelling results of Vollset *et al*. (2021) reflect this observation, in which studies estimating the outmigration timing of smolts were found to estimate earlier overall outmigration timing than studies using video or trap methods that are not size selective in the way that tagging is. When using electronic tagging to estimate the timing of smolt migrations, the smallest possible tags should be used so that the full range of the smolt size distribution can be tagged and monitored through the season. Presently, the smallest smolt tags are open‐source Juvenile Salmon Acoustic Telemetry System (JSATS) tags, measuring 12 mm long and 0.08 g in air with a battery life of 30 days and transmitting at 417.6 kHz (Deng *et al*., 2021).

Atlantic salmon smolts grew throughout the study and the growth differed among treatment groups. The most important finding of this study was that dummy acoustic transmitters exhibited some evidence of impairing the growth of smolts during the study, albeit not significant. The dummy acoustic transmitter had the largest volume of any tag in this study by far, and may restrict the potential stomach fullness of the smolts. Some of the variation in growth for the sham and dummy tagged groups was captured by the differences in initial length because the dummy tagged fish were longer to begin with. Additional variance, related to initial length, was captured by the time interval between tagging and recapture; shorter times to recapture the larger dummy tagged fish contributed in part to their lesser growth. Nevertheless, there was some evidence that smolts surgically implanted with dummy transmitters grew less than counterparts in the study. Surgeries are longer and require more handling than simple PIT implantation *via* ventral incision, which may affect the overall recovery trajectory of the animal. Larsen *et al*. (2013) found that larger PIT tags did not affect the growth of similar‐sized Atlantic salmon in a laboratory setting. Lacroix *et al*. (2004) found growth of Atlantic salmon tagged with similar acoustic tags had slower initial growth that eventually caught up to untagged counterparts. Ours is a unique effort to document growth in free swimming and wild salmon *in situ* and suggests some hindrance on growth that should be acknowledged when using instrumented salmon smolts to understand population‐level processes such as migration timing.

Overall, the dummy acoustic transmitters in this study performed well and the results support the use of acoustic tags as a tool for monitoring Atlantic salmon smolt migrations, with the necessary caveats. The dummy transmitters in this study were 14.5 mm long, which was 10.5% ± 0.44% of the body length for dummy tagged salmon smolts. A meta analysis of tag length for juvenile salmonids revealed increasing mortality when tags were beyond 17.5% of the fish's total length (Vollset *et al*., 2020). Tag weight was 1.2 g in air, on average 5.2% ± 0.50% of the body weight. The rule of thumb in fish telemetry is for tags to weigh <2% of body weight in air, but this is often violated for salmon smolts due to challenges procuring tags sufficiently light for fish weighing 15–30 g. In practice, heavier tag burdens are common for salmon smolts (Newton *et al*., 2016). Brown *et al*. (1999) showed that tags 6%–12% of body mass did not affect the swimming performance of rainbow trout (*Oncorhynchus mykiss* Walbaum 1792) and Berhe (2021) suggested that fish having tag burdens about 8%–12% of body weight had normal diel vertical patterns of depth use in a holding study of Atlantic salmon smolts. Moore *et al*. (1990) used dummy transmitters weighing 1.3 g in air (weight of fish not reported but lengths were 12.2–18.9 cm) and observed some delayed growth of tagged fish, but Robertson *et al*. (2003) studied juvenile Atlantic salmon in Newfoundland, Canada, and showed that food consumption was similar between tagged parr and untagged counterparts. Newton *et al*. (2016) resolved no effect of smolt size on survival of 68 salmon smolts in an Irish river. The survival of dummy tagged smolts with 4%–6% tag burden not being significantly different from that of PIT tagged counterparts with a less invasive surgery and much smaller sized tag implanted provides support that the effects of the acoustic tags are minimal during freshwater migration.

Despite weak evidence for differences among the tags, we are cautious about the interpretation of these results given our logistic regression had only a 20% chance of detecting a 10% difference in migration and a 58% chance of detecting a true 20% difference in migration of the dummy tagged fish. A sample of about 500 fish per group would be required to achieve >95% likelihood of detecting a 10% difference in smolt survival and our study may be integrated into future meta‐analyses striving to identify tag effects. We were limited in our ability to procure 500 smolts per group by logistical constraints, including the small size of the Dale River making such numbers unattainable. Many acoustic tagging studies are limited by sample size and our efforts to emulate such studies and evaluate migration, timing and growth compared to sham surgeries and PIT tagged fish resulted in some gaps. The finding that the dummy tagged salmon and 16 mm PIT tagged salmon had similar migration suggests that these two groups had similar performance, but less migration among smaller smolts explains some of the difference.

Our study design was not a fully factorial design. An effective design for analysis purposes would have been to have surgery and tag types as two factors such that there was a surgically implanted dummy transmitter and a surgically implanted PIT tag (*i.e*., sham as done in our study) compared with a PIT tag inserted through a small nonsurgical incision (*i.e*., as in our study) and a dummy transmitter inserted through a small nonsurgical incision. It is not possible to insert a dummy transmitter through a nonsurgical incision, which is why we were not able to conduct such an idealistic factorial study. A future investigation may, however, modify a PIT tag with tungsten or another heavy material to match the weight of the dummy acoustic tag and achieve a more factorial design comparing implantation method (surgery or small incision) with tag weight (0.1 or 1.2 g). The sham surgery is the closest we can come to a true control in this field setting and provided good evidence to separate the effects of the surgical procedure from the tag burden and compare with a less invasive method of PIT tag implantation. We also added an additional treatment for comparison, the 16 mm PIT tag, which is a new intermediate size of PIT tag that may be useful where 12 mm tags are limited by the detection range and where 23 mm tags are too large. We were not able to add an additional group to compare these groups to a 23 mm PIT tagged group because the fish in the Dale River were too small for such a long tag. Fortunately, the results obtained suggest that the 16 mm PIT tag had good performance compared to the 12 mm tag, supporting its use for applications where 23 mm tags are too large but 12 mm tags are undesirable due to lower detectability. Although our study design was not fully factorial, comparing the migration for fish in the four treatment groups yielded usable results.

## 5 CONCLUSIONS

Managers must interpret scientific results in the context of the experimental design, and may put less emphasis on telemetry results if there is an impression that the results are biased by tagging effects. Our study shows differences in the migration behaviour of fish for different tagging treatments. The results are favourable both for the use of 16 mm PIT tags as an alternative to 12 mm PIT tags as well as the use of acoustic transmitters with tag burden 4%–6% of the body weight for certain research and monitoring applications. Managers can be confident that the results of acoustic studies conducted with such tags yield representative results compared to the migration of other tagged smolts but should be aware that the migration timing will only be representative of the size classes tagged, missing the later migrating smaller fish and yielding the illusion of a shorter smolt migration period. Applying these results to making tagging decisions, investigators should consider how size limitations of using surgically implanted acoustic tags will affect the growth and probability of successful migration of tagged fish relative to counterparts. Development and establishment of micro‐acoustic transmitters (*e.g*., Deng *et al*., 2021) for studying smolts seems to be an important frontier in this field for generating the most reliable data on migrating smolts.

## AUTHOR CONTRIBUTION

R.J.L., E.S., T.H. and K.W.V. conceived and designed the study. R.J.L., E.S., T.W., E.M.H. and K.W.V. implemented the study. R.J.L., L.S.D., T.H., E.S. and K.W.V. analysed data and wrote the initial draft. All authors contributed to finalization of the draft and approve manuscript submission.

## ETHICS STATEMENT

The care and use of experimental animals complied with Norway's animal welfare laws, guidelines and policies as approved by Mattilsynet protocol number 27407.

## Supporting information


**Appendix S1** Supporting Information

## Data Availability

Data were collected by NORCE Norwegian Research Centre AS. The authors and institution are committed to open data and upon publication the data will be made available at www.zenodo.org.
