# Supplementary material for: Effects of tag type and surgery on migration of Atlantic salmon (Salmo salar) smolts
Source: J Fish Biol. 2022 Jun 27;101(3):515–21. doi: 10.1111/jfb.15116 (PMC9545663; doi:10.1111/jfb.15116)
Supplement: Supplementary file 1 — Appendix S1 Supporting Information [file JFB-101-515-s001.pdf]

Supplement

For those interested, we conducted our power analysis using the following code:

```
## power analysis
set.seed(65)
f<-function(x) {glm(sur~treat, family="binomial",
                    data=tibble(treat=rep(unique(sur$treat), 50),
                                sur=rbinom(n=50*4, size=1,
                                           prob=c(0.8, 0.8, 0.7, 0.8)))) %>%
    broom::tidy()}

pow<-purrr::map(seq_len(10000), ~f(1)) %>%
  bind_rows() %>%
  dplyr::filter(term!="(Intercept)")

pow %>%
  ggplot(aes(p.value, fill=term))+
  geom_density(alpha=0.5)

pow %>%
  mutate(sig=case_when(p.value<0.05~ "sig", T~"ns")) %>%
  group_by(term, sig) %>%
  count %>%
  pivot_wider(names_from=sig, values_from=n) %>%
  mutate(p=sig/(ns+sig))
```
